# Epigenetically driven impairment of BDNF–ARC signaling contributes to circadian and cognitive disarray in a mouse model of postoperative delirium

**DOI:** 10.1002/alz.71556

**Published:** 2026-06-15

**Authors:** Hari Prasad Osuru, Navya Atluri, Tam Le, Jinny Park, Michal Jedrusiak, Elzbieta Dulko, Meghana Illendula, Nadia Lunardi

**Affiliations:** ^1^ Department of Anesthesiology University of Virginia Health Charlottesville USA; ^2^ Visiting Scholar Centre for Intensive Care and Perioperative Medicine Jagiellonian University Medical College Krakow Poland; ^3^ Neuroscience Graduate Program University of Virginia Charlottesville USA

**Keywords:** activity‐regulated cytoskeleton‐associated protein (ARC) protein, brain‐derived neurotrophic factor (BDNF)‐ tropomyosin receptor kinase B (TrkB) signaling, circadian locomotor output cycles protein kaput (CLOCK) genes, cognitive‐dementia risk, dendritic plasticity, epigenetic repression, postoperative delirium

## Abstract

**INTRODUCTION:**

Postoperative delirium (POD) is common in older surgical patients and is clinically associated with an increased risk of long‐term cognitive decline and dementia; disrupted BDNF signaling and circadian dysregulation are implicated, but their coordinated mechanisms remain unclear.

**METHODS:**

Aged male C57BL/6J mice were exposed to anesthesia, surgery, and intensive care unit‐like stress (ASI). Hippocampal neuroplasticity, dendritic morphology, epigenetic regulation, and circadian signaling were assessed using molecular assays, imaging, and protein–protein interaction (PPI) network analysis.

**RESULTS:**

ASI reduced brain‐derived neurotrophic factor (BDNF)–activity‐regulated cytoskeleton‐associated protein signaling, dendritic structural complexity, and attention, with accompanying histone hypoacetylation, an increased 5‐methylcytosine/5‐hydroxymethylcytosine ratio, and disruption of network hubs centered on BDNF and circadian regulators. Suberoylanilide hydroxamic acid mitigated these effects and improved short‐term cognitive performance.

**DISCUSSION:**

Perioperative stress is associated with an epigenetically repressed, synaptically impaired hippocampal state linked to delirium‐like behavior and cognitive vulnerability. Targeting chromatin accessibility and BDNF–circadian coupling with histone deacetylase inhibition may mitigate acute cognitive consequences following surgery.

## Highlights

- ASI suppresses hippocampal BDNF–TrkB–CREB–ARC signaling and promotes dendritic structural alterations in aged mice.
- Perioperative stress is associated with epigenetic dysregulation characterized by histone hypoacetylation and increased DNA methylation‐linked transcriptional repression.
- Network analysis identifies BDNF and core CLOCK genes as convergent hubs linking synaptic and circadian disruption.
- HDAC inhibition mitigates epigenetic dysregulation, restores synaptic structure, and attenuates delirium‐like cognitive deficits.
- Perioperative epigenetic and synaptic alterations may intersect with biological pathways of cognitive vulnerability and potential risk for long‐term cognitive decline and dementia.

## 1 BACKGROUND

Postoperative delirium (POD) is an acute neurocognitive disorder frequently observed in intensive care unit (ICU) patients following anesthesia and surgery.^1^ It is characterized by disturbances in attention, awareness, and cognition that typically emerge within hours to days after surgical intervention.^2^ Even transient episodes of POD are associated with serious adverse outcomes, including falls, aspiration, respiratory failure, and prolonged mechanical ventilation.^3^ Older adults are particularly vulnerable and prone to extended hospitalizations, increased morbidity, and elevated mortality.^4^ The American Delirium Society estimates that delirium affects approximately seven million hospitalized Americans annually, contributing $143 to $152 billion in healthcare costs, with older surgical patients accounting for $26 to $42 billion each year.^5^, ^6^ Importantly, POD is one of the strongest clinical risk indicators for long‐term cognitive decline and dementia.^7^

Despite its clinical impact, POD mechanisms remain poorly defined, and no US Food and Drug Administration (FDA)‐approved therapies exist. Prior emphasis on downstream processes such as neuroinflammation and oxidative stress has yielded limited benefit, implying that earlier drivers are critical. Emerging evidence points to acute, reversible synaptic dysfunction as a central feature of delirium, yet upstream molecular events precipitating perioperative synaptic failure remain unclear.

To address this gap, our laboratory established a translational aged C57BL/6J mouse model for POD using anesthesia, surgery, and ICU‐like stress (ASI).^8^ This model recapitulates core delirium features, including attention and memory impairments, sleep fragmentation, and circadian dysregulation.^9^ At the molecular level, ASI is associated with suppression of synaptic plasticity‐related gene and protein networks,^8^, ^10^ providing an experimental framework to examine mechanisms by which perioperative stress may contribute to synaptic vulnerability and cognitive decline risk.

Dendritic degeneration and spine loss are hallmark features of aging and early neurodegenerative disease and strongly correlate with cognitive decline.^11^ Brain‐derived neurotrophic factor (BDNF) is abundantly expressed in the adult hippocampus and plays a central role in maintaining dendritic architecture, synaptic strength, and memory consolidation.^12^, ^13^ Binding of BDNF to its high‐affinity receptor, Tropomyosin receptor kinase B (TrkB), activates downstream Mitogen‐Activated‐Protein Kinase/Extracellular‐Signal‐Regulated Kinase (MEK/ERK) signaling cascades that converge on phosphorylation of cyclic AMP response element binding protein (CREB) and induction of activity‐regulated cytoskeleton‐associated protein (ARC), a key regulator of synaptic remodeling and long‐term memory formation.^14^, ^15^, ^16^ Notably, dysregulation of the BDNF–TrkB–CREB–ARC signaling axis has been implicated in neurodegenerative disease like Alzheimer's disease (AD) and Parkinson's disease.^17^, ^18^, ^19^

Emerging evidence indicates that neuroplastic signaling is tightly coupled to circadian regulation. BDNF expression and synaptic remodeling exhibit circadian oscillations coordinated by circadian locomotor output cycles protein kaput (CLOCK) and basic helix‐loop‐helix ARNT‐like (BMAL1) transcriptional programs that gate activity‐dependent plasticity across sleep–wake cycles.^20^, ^21^, ^22^ Circadian disruption is a defining feature of POD in the ICU and is also prevalent in AD, manifesting as sleep fragmentation, sundowning, and impaired cognitive homeostasis.^23^ These shared features suggest that delirium and dementia may converge on molecular vulnerabilities involving disrupted coupling of neuroplastic and circadian transcriptional programs.

Epigenetic regulation represents a compelling upstream mechanism that may link perioperative stress to coordinated transcriptional failure. DNA methylation and histone acetylation dynamically control chromatin accessibility at neuroplasticity‐ and circadian‐related loci, including BDNF and CLOCK. Anesthesia, surgery, and critical illness induce histone hypoacetylation and aberrant DNA methylation, suppressing CREB‐dependent transcription and impairing memory formation.^24^, ^25^ Such epigenetic repression is associated with synaptic dysfunction, sleep disruption, and cognitive decline.^26^, ^27^, ^28^, ^29^ However, whether these epigenetic mechanisms concurrently disrupt neurotrophic and circadian transcription in POD remains unexplored.

In this study, we tested whether perioperative stress was associated with epigenetic alterations in the hippocampus that disrupt coordinated BDNF and circadian CLOCK signaling. Building on evidence that histone deacetylase (HDAC) inhibitors exert neuroprotective effects across neurological and neurodegenerative conditions, we examine whether restoring histone acetylation mitigates synaptic, circadian, and delirium‐like behavioral deficits. By integrating molecular, structural, and behavioral analyses, this work positions perioperative epigenetic dysregulation as a potential contributor to POD and cognitive vulnerability along the delirium–dementia continuum. Importantly, this study is intended to provide mechanistic insight into vulnerability pathways rather than direct evidence of progression to dementia.

**RESEARCH IN CONTEXT**

1. **Systematic review**: Few clinical and preclinical studies have implicated reduced BDNF signaling, sleep–wake disruption, and cognitive disorganization in POD. However, the integrated contributions of epigenetic dysregulation, neurotrophic signaling failure, and circadian dysfunction remain poorly defined.
2. **Interpretation**: ASI is associated with an epigenetically repressed hippocampal state that disrupts coordinated BDNF–ARC signaling and circadian transcriptional networks. This combined disruption is accompanied by dendritic alterations and delirium‐like cognitive impairment. By identifying chromatin dysregulation as a mechanistic nexus linking synaptic vulnerability and circadian disorganization, these findings provide a biological framework that may relate acute POD to increased cognitive vulnerability and long‐term cognitive decline and dementia.
3. **Future directions**. We will employ precision single‐cell multi‐omics approaches to map hippocampal cell‐type‐ and gene‐specific epigenetic alterations associated with POD vulnerability. Integration of scATAC‐seq, scCUT&Tag‐seq, single‐cell DNA methyl‐seq, and scNMT‐seq will enable assessment of BDNF‐ and CLOCK‐locus chromatin accessibility and its relationship to transcriptional output across CA1 neurons, microglia, and astrocytes. These analyses will define cell‐specific epigenetic signatures relevant to synaptic and circadian regulation and provide a framework for future studies of perioperative stress‐induced delirium and biological pathways associated with cognitive vulnerability and potential risk for cognitive decline and dementia.

## 2 METHODS

**FIGURE 1:**
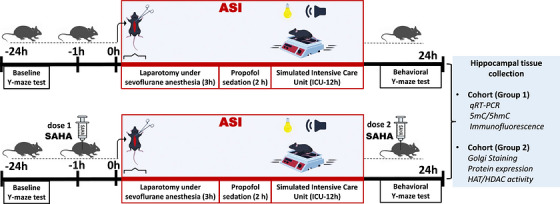
Experimental design of the anesthesia–surgery‐simulated ICU (ASI) model showing ASI exposure, SAHA treatment, behavioral testing, hippocampal tissue collection, and subsequent molecular and histological analyses.

All animal procedures were approved by the University of Virginia Institutional Animal Care and Use Committee (Charlottesville, VA, USA). In this study, 18‐ to 20‐month‐old C57BL/6J male mice (Jackson Laboratory, USA) were randomly assigned to either the ASI group or the control group. The ASI protocol was performed as previously described.^8^ Briefly, mice in the ASI group underwent laparotomy under sevoflurane anesthesia for 3 h, followed by 2 h of sedation with propofol. Subsequently, mice were exposed to simulated ICU conditions for 12 h, which included intermittent light exposure, noise, and cage shaking. Mice in the control group did not undergo the ASI protocol. To modulate ASI‐induced epigenetic alterations, a subset of mice was treated with the HDAC inhibitor vorinostat (suberoylanilide hydroxamic acid [SAHA]). Details of SAHA administration and associated experimental procedures are described in Methods Section 2.6, and a schematic overview of the experimental design and timeline is provided in Figure 1.

The experiments in this study were conducted using two independent cohorts (Group 1 and Group 2) of aged mice, due to limitations in hippocampal tissue volume and mutually exclusive processing requirements for molecular versus histological analyses. Both cohorts were exposed to identical ASI conditions and behavioral testing paradigms, and delirium status was determined using Z‐score‐based thresholds established in our previous delirium animal model validation study.^8^ All tissues were processed in parallel using standardized laboratory protocols to minimize technical variability.

Group 1 animals comprised a cohort used during initial delirium animal model validation. Behavioral outcomes from this cohort were previously published by our laboratory.^8^ Real‐time quantitative polymerase chain reaction (qRT‐PCR), global DNA methylation (5‐methylcytosine [5mC]/5‐hydroxymethylcytosine [5hmC]), and immunofluorescence analyses reported here were generated from this cohort's brain tissue. Group 2 animals were used for behavioral, Golgi, protein, and epigenetic (histone acetyltransferase [HAT]/HDAC activity) experiments across control, ASI, SAHA, and SAHA+ASI groups, including HDAC1‐3 protein expression analyses. Experimental group allocation and animal numbers for each assay are summarized in Table S1. All experiments were conducted in aged male mice, in continuity with our previously validated ASI delirium model, to ensure direct comparability across behavioral, molecular, and epigenetic endpoints.

### 2.1 Y‐Maze novel arm preference test

Cognitive function was assessed using the Y‐maze novel arm preference test 24 h after ASI (*n* = 15 mice per group). The protocol was adapted from our previous studies.^8^ The Y‐maze apparatus consisted of three identical arms (35 × 35 × 35 cm). The test comprised two trials. During the first (training) trial, the start arm and one familiar arm were left open, while the third (novel) arm was blocked. Mice were placed in the start arm and allowed to freely explore the open arms for 5 min. After a 60‐min inter‐trial interval, the second (testing) trial was conducted, in which all three arms were opened. Mice were again placed in the start arm and allowed to explore the maze for 5 min. The number of entries into the novel arm during the testing trial was recorded and used as an index of spatial recognition memory and attention. Delirium‐like behavioral status was classified using Z‐score‐based thresholds established in our prior delirium animal model validation study.^8^ Baseline behavioral testing was performed 24 h prior to ASI exposure. Behavioral testing was repeated 24 h after ASI initiation, following recovery of the righting reflex, spontaneous locomotion, and baseline grooming and feeding behaviors to minimize potential confounding effects of residual anesthesia or sickness behavior.

Our group previously established and validated an anesthesia, surgery, and ICU model for delirium using a multidimensional behavioral battery (including Y‐maze, open‐field, and buried food tests) to assess complementary domains of delirium‐like behavior such as attention, exploratory behavior, and motivation.^8^ However, in this mechanistic study, the need for time‐sensitive brain collection at 24 h after ASI limited the feasibility of performing a full behavioral battery. Within these constraints, we selected the Y‐maze based on its consistent sensitivity and reproducibility in our prior validation studies, as well as its ability to detect hippocampal‐dependent deficits in spatial working memory. This task provides an ethologically relevant, low‐stress measure of exploratory behavior^30^, ^31^ and reliably captures the behavioral phenotype associated with ASI.^30^, ^32^ Z‐scores were calculated by subtracting post‐ASI performance from baseline values and normalizing to the standard deviation of the control group, with negative values indicating deterioration. A Z‐score equal to or less than −1.96 was used to define delirium‐like behavior, as previously described.^8^

### 2.2 Golgi‐Cox staining

We performed Golgi‐Cox staining (*n *= 5 per group) to examine neuronal and glial morphology in hippocampal sections obtained from control and ASI mice following the Y‐maze behavioral test. Mice were transcardially perfused with 0.1 M phosphate‐buffered saline (PBS) to collect brain tissue, which was then processed using the FD Rapid GolgiStain Kit (Cat#PK401, FD NeuroTechnologies) following the manufacturer's instructions. After immersion in Golgi reagents, brains were sectioned at 150‐µm thickness through the hippocampus using a vibratome. The sections were subsequently stained and imaged using a Leica Thunder microscope (model DMi8, Leica Microsystems, Deerfield, IL, USA). Tile scans of the hippocampal CA1 region were acquired under brightfield mode with z‐stacks at 63× magnification. Image analysis was performed using ImageJ software. For each animal, five structurally intact and well‐impregnated neurons were randomly selected. The following parameters were quantified: (1) the average number of dendritic branches from the longest apical dendrite, (2) the length of the longest apical dendrite originating from CA1 pyramidal neurons, and (3) dendritic spine density in the CA1 region. These metrics were compared between ASI and control mice to assess neuroplastic alterations associated with the ASI condition.

### 2.3 Hippocampal gene and protein expression analysis

Following completion of behavioral testing, total hippocampi were rapidly dissected from whole brains and immediately stored in liquid nitrogen or at −80°C for subsequent gene and protein expression analyses. All epigenetic and transcriptional analyses were performed on whole hippocampal tissue homogenates. Although delirium is recognized as a network‐level disorder involving prefrontal, hippocampal, and subcortical circuits,^33^, ^34^ the hippocampus was selected as an initial region of interest due to its central role in attention and memory.^35^, ^36^

#### 2.3.1 Target gene expression—qRT‐PCR

Total RNA was isolated from pooled hippocampal tissue samples of control and ASI mice (*n *= 3 independent biological pools per group; behavioral classification was established at the individual‐animal level prior to tissue pooling, and pools were generated exclusively from animals with concordant behavioral phenotypes) using the RNeasy Plus Mini Kit (Qiagen, Cat#74134). A pooled qRT‐PCR approach was employed, as previously described,^8^, ^9^, ^37^ to enable sufficient RNA input for gene expression profiling in aged hippocampal tissue following ASI and to reduce technical variability across low‐input samples, consistent with prior screening approaches in this model.^8^, ^9^

For gene expression analysis, 1 µg of total RNA was reverse‐transcribed using the iScript cDNA Synthesis Kit (Bio‐Rad). Complementary DNA (2 µL) was subsequently subjected to real‐time quantitative PCR using the CFX Connect Real‐Time PCR System (Bio‐Rad) in a final reaction volume of 20 µL containing 1× SsoAdvanced SYBR Green Supermix and forward and reverse primers (predesigned primers from Sigma‐Aldrich or Bio‐Rad; sequences presented in Table S2). All reactions were performed in technical triplicate. Gene expression levels were normalized to the reference gene Gapdh, and relative expression was calculated using the 2^−ΔΔCt method.^38^ Fold changes (FCs) were expressed relative to control samples.

Because tissues were pooled prior to RNA extraction, statistical analyses were performed on biological pool replicates (*n* = 3 per group) rather than individual animals. Accordingly, statistical comparisons were performed between independent biological pool replicates using Student's *t*‐test to compare group‐level differences, with *p* < 0.05 considered statistically significant. To corroborate mRNA findings, protein expression of selected differentially regulated genes was evaluated by Western blot analysis.

#### 2.3.2 Protein expression level quantification – Western blotting

Total protein was extracted from hippocampal tissue of control and ASI mice (*n *= 5 per group) as previously described.^8^, ^9^ In brief, tissues were lysed in radioimmunoprecipitation assay (RIPA) buffer (Thermo Fisher Scientific, Cat#89901), and protein concentrations were determined using the Pierce BCA Protein Assay Kit (Thermo Fisher Scientific, Cat#23227). Whole‐tissue lysates (10 to 30 µg) were separated on 4% to 20% gradient SDS‐PAGE gels (Bio‐Rad) and transferred onto PVDF membranes (Millipore). Membranes were blocked with SuperBlock T20 (PBS) blocking buffer (Thermo Fisher Scientific, Cat#37515), followed by overnight incubation at 4°C with primary antibodies against target proteins (see Table S3 for antibody details and dilutions). Following washes with Tris‐buffered saline containing 0.1% Tween‐20 (TBST), membranes were incubated with appropriate Horseradish Peroxidase (HRP)‐conjugated secondary antibodies for 1 h at room temperature. Immunoreactive bands were visualized using chemiluminescent substrate (SuperSignal West; Thermo Fisher Scientific, Cat#34096) and imaged using a G:BOX gel documentation system (Syngene). Densitometric analysis was performed using GeneTools software (Syngene). Protein expression levels were normalized to β‐tubulin (loading control) and expressed as percent change relative to control samples.

### 2.4 Immunofluorescent FlexAble staining

Immunofluorescent staining was performed to assess hippocampal localization of BDNF, CREB‐binding protein (CBP), and ARC as previously described with minor modifications.^8^, ^39^ Following behavioral testing, mice were transcardially perfused with 4% paraformaldehyde (PFA), and brains were collected, paraffin‐embedded, and sectioned at 5 µm using a microtome (Research Histology Core, University of Virginia).

Sections were deparaffinized, rehydrated, subjected to heat‐induced antigen retrieval, permeabilized for 20 min, and blocked with 5% normal goat serum containing 1% BSA for 2 h at room temperature. Sections were then incubated overnight at 4°C with FlexAble CoraLite Plus‐conjugated primary antibodies against BDNF, CBP, or ARC, together with NeuN (neuronal marker) or Iba1 (microglial marker) for multiplex cellular localization analyses (primary antibodies were conjugated using the Proteintech FlexAble CoraLite Plus Antibody Labeling Kit with fluorophores 488, 555, 647, or 405 according to the manufacturer's instructions). Antibody details are provided in Table S3.

Multiplex staining was performed to identify target protein expression within neuronal and microglial populations. Nuclear counterstaining was omitted in multiplex experiments to avoid spectral channel limitations. In parallel, separate sections were stained for BDNF, CBP, or ARC and counterstained with Hoechst 33342 (1:1000; Thermo Fisher Scientific) to visualize regional hippocampal distribution and CA1 cytoarchitecture.

Slides were mounted with Fluoromount‐G (Southern Biotech) and imaged at 20× magnification using a Leica Thunder microscope (DMi8, Leica Microsystems) at the University of Virginia Advanced Microscopy Facility. For cell‐specific expression analyses, double‐positive cells (target protein^+^/cell‐marker^+^ combinations; NeuN for neurons and Iba1 for microglia) were identified within the hippocampal CA1 region and quantified as cell density (cells/µm^2^) using ImageJ software (US National Institutes of Health). Images from Hoechst‐counterstained sections were used to assess regional target protein distribution within the CA1 hippocampal subfield and were quantified as positive cells per unit area (cells/µm^2^). Image acquisition parameters were kept constant across groups to ensure comparability. Immunofluorescence analyses were performed using *n* = 4 mice per protein.

### 2.5 Assessment of hippocampal epigenetic changes

#### 2.5.1 Histone acetylation (HAT and HDAC) activity‐level quantification by ELISA

HAT and HDAC activities in hippocampal tissue were quantified using enzyme‐linked immunosorbent assay (ELISA) kits, as previously described.^40^ Total nuclear protein was extracted from the hippocampus (*n* = 4 to 5 per group), and 10 µg of nuclear extract was used per assay. HAT activity was measured using the Epigentek HAT Activity Assay Kit (Cat#P‐4003), and HDAC activity was measured using the HDAC Activity Assay Kit (Cat#P‐4034), both from Epigentek (New York, USA). Assays were performed according to the manufacturer's protocols using a colorimetric detection method. Absorbance was read at 450 nm using a microplate reader (Bio‐Rad, USA). Enzyme activity levels were calculated according to kit instructions and expressed as percent change relative to control samples. The HAT/HDAC activity ratio was calculated to assess the balance between histone acetylation and deacetylation.

#### 2.5.2 DNA methylation (5mC and 5hmC) level quantification by ELISA and dot blot

Global DNA methylation and hydroxymethylation levels in the hippocampus were assessed by quantifying 5mC and 5hmC using both ELISA and dot‐blot‐based approaches.

**ELISA‐based quantification**: Genomic DNA was extracted from whole hippocampal tissue using the DNeasy Blood & Tissue Kit (Cat#69504, Qiagen) and quantified using a NanoDrop spectrophotometer (Thermo Fisher Scientific). For each sample, 50 ng of DNA was used to measure 5mC levels using the MethylFlash Global DNA Methylation (5mC) ELISA Easy Kit (Cat#P‐1030, Epigentek) and 5hmC levels using the MethylFlash Global DNA Hydroxymethylation (5hmC) ELISA Easy Kit (Cat#P‐1032, Epigentek). Assays were performed according to the manufacturer's instructions with colorimetric detection, and absorbance was measured at 450 nm. Percent changes in 5mC and 5hmC, as well as the 5mC/5hmC ratio, were calculated relative to control values to evaluate the balance between DNA methylation and active demethylation. ELISA‐based quantification (*n* = 3 per group) provides sensitive quantitative measurement of global 5mC and 5hmC levels, while dot blot analysis (*n* = 6 per group) serves as an independent validation approach in an expanded cohort.

**Dot blot analysis of 5mC and 5hmC**: Dot blot analysis was performed as previously described.^41^, ^42^ Briefly, 1 µg of genomic DNA (*n* = 6 mice per group) was denatured at 95°C for 10 min, immediately cooled on ice, and spotted onto a positively charged nylon membrane (Millipore Sigma). Membranes were air‐dried for 20 min and ultraviolet‐light‐crosslinked (120 mJ/cm^2^), then blocked with SuperBlock (PBS) blocking buffer (Thermo Fisher Scientific, Cat#37515) for 1 h at room temperature. Membranes were incubated overnight at 4°C with primary antibodies against 5mC or 5hmC (antibody details provided in Table S3), followed by incubation with HRP‐conjugated secondary antibodies. Signals were developed using enhanced chemiluminescence (ECL) and imaged using a G:BOX gel documentation system (Syngene).

DNA loading was verified by post‐staining membranes with methylene blue. Densitometric quantification of 5mC and 5hmC dot intensities was performed and normalized to the corresponding methylene blue loading signal. Percent changes relative to control and the 5mC/5hmC ratio were calculated as indices of the balance between DNA methylation and active DNA demethylation.

### 2.6 HDAC activity inhibition with SAHA

SAHA (vorinostat), a FDA‐approved pan‐HDAC inhibitor primarily targeting class I, II, and IV HDACs, was used to modulate ASI‐induced epigenetic changes. Mice were administered SAHA intraperitoneally (i.p.) at a dose of 50 mg/kg (Selleck Chemicals, USA), formulated in 10% dimethyl sulfoxide in normal saline. This dose and regimen were selected based on prior in vivo studies demonstrating effective HDAC inhibition and increased histone acetylation in several neurodegenerative and neurological disorders.^43^, ^44^, ^45^, ^46^ Mice received the first dose 1 h prior to ASI and a second dose 12 h after ICU.

Following completion of behavioral testing, hippocampal tissue was collected for downstream molecular and neuronal morphology analyses. Histone acetylation activity (HAT and HDAC), protein expression of key regulators of dendritic architecture, synaptic plasticity, memory, and circadian signaling (BDNF, ARC, and CLOCK), class I HDAC isoforms (HDAC1‐3), and Golgi‐Cox neuronal morphology were evaluated using the procedures described earlier.

### 2.7 Protein–protein interaction (PPI) and hub gene network analysis

PPI networks were constructed and analyzed using the STRING database (version 12.0; https://string‐db.org/).^47^ A total of 15 proteins involved in BDNF/TrkB signaling (BDNF, NTRK1‐3, CREB1, CREBBP, ARC, c‐FOS) and core circadian clock components (CLOCK, ARNTL, PER1‐3, CRY1‐2) were submitted to STRING to identify known and predicted interactions using a high‐confidence interaction score threshold of ≥0.7. Network visualization and functional enrichment analyses were performed within the STRING interface. To identify highly connected nodes within the network, the resulting interaction network was imported into Cytoscape, and the top 10 hub genes were identified using the CytoHubba plugin.^48^

### 2.8 Statistical analysis

Statistical comparisons between two groups were performed using unpaired, two‐tailed *t*‐tests for gene and protein expression, Golgi‐Cox staining, and Y‐maze analyses. Comparisons among SAHA, ASI, and SAHA+ASI groups were conducted using ordinary one‐way analysis of variance (ANOVA), followed by Tukey's multiple‐comparisons test. All statistical analyses were performed using GraphPad Prism 9 (GraphPad Software, USA). Data are presented as mean ± SEM, with significance levels set at *p < 0.05, **p < 0.01, ***p < 0.001, and ****p < 0.0001. Sample sizes were determined based on prior studies from our laboratory and published literature examining epigenetic and neuroplasticity endpoints in aged rodent models.^8^, ^9^, ^49^ Experimental group allocation and assay‐specific animal numbers are summarized in Table S1.

## 3 RESULTS

### 3.1 ASI suppresses transcription of neuroplasticity and BDNF pathway genes

**FIGURE 2:**
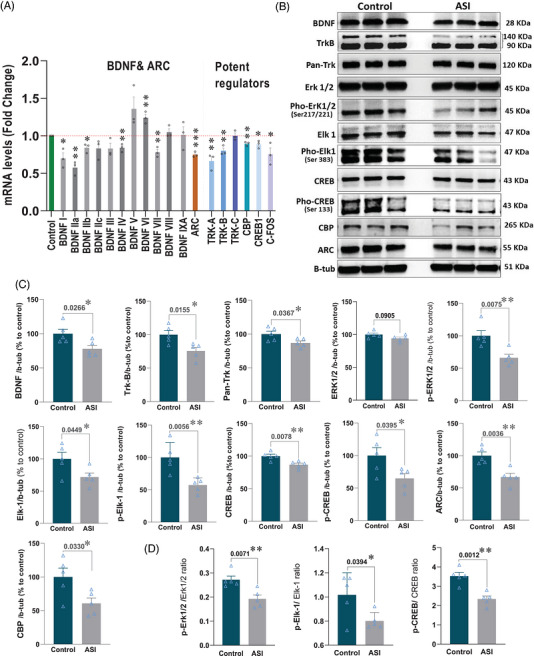
ASI alters hippocampal BDNF–Trk–CREB–ARC signaling at transcriptional and protein levels. (A) Hippocampal mRNA expression of BDNF exons (I–IXA), ARC, TrkA, TrkB, TrkC, c‐FOS, CREB1, and CREB‐binding protein (CREBBP) in Control and ASI mice (*n* = 3 animals per group). (B–D) Protein expression of BDNF, TrkB, pan‐Trk, ERK1/2, phospho‐ERK1/2, ELK1, phospho‐ELK1, CREB, phospho‐CREB, CBP, and ARC, including representative immunoblots (B), densitometric quantification normalized to β‐tubulin and expressed as percent change relative to Control (C), and ratios of phosphorylated to total ERK1/2, ELK1, and CREB (D) (*n* = 5 Control and *n* = 5 ASI mice). Data are presented as mean ± SEM. Statistical comparisons were performed using two‐tailed unpaired *t*‐tests. *p < 0.05, **p < 0.01.

Hippocampal qRT‐PCR mRNA expression profiling revealed coordinated transcriptional dysregulation of BDNF pathway‐related genes in ASI‐exposed aged mice compared with controls (Figure 2A). Values represent FC relative to control animals. Expression of multiple BDNF transcripts (exons I–IXA) was significantly altered. Specifically, BDNF exon I (0.69, *p* = 0.01), exon IIa (0.57, *p* = 0.001), exon IIb (0.84, *p* = 0.01), exon IV (0.84, *p* = 0.006), and exon VII (0.78, *p* = 0.004) transcripts were significantly reduced. In contrast, BDNF exon VI transcripts were significantly increased (1.24, *p* = 0.005), whereas BDNF exons IIc, III, V, VIII, and IXA did not show significant changes.

Similarly, several downstream neuroplasticity pathway genes were significantly downregulated, including Arc (0.75, *p* = 0.003), Ntrk1 (TrkA; 0.66, *p* = 0.006), Ntrk2 (TrkB; 0.80, *p* = 0.005), Creb1 (0.88, *p* = 0.01), Crebbp (CBP; 0.89, *p* = 0.002), and FOS (c‐Fos; 0.75, *p* = 0.04), while Ntrk3 (TrkC) expression remained unchanged. Notably, BDNF exon IV and exon VI transcripts, which represent activity‐dependent BDNF variants associated with synaptic plasticity and circadian regulation, were among the most prominently dysregulated following ASI exposure. Together, these transcriptional alterations are consistent with the disruption of activity‐dependent neurotrophic signaling, representing an early molecular response associated with POD vulnerability.

### 3.2 Protein analysis confirms loss of BDNF signaling capacity and CREB–ARC activation

Western blot analysis (Figure 2B) with densitometric quantification (Figure 2C) revealed significant reductions in multiple components of the BDNF signaling cascade in ASI hippocampi compared with controls. Protein levels of BDNF (−22.1 ± 8.1%, *p* = 0.02), TrkB (−24.4 ± 7.9%, *p* = 0.01), Elk‐1 (−28.2 ± 11.9%, *p* = 0.04), CREB (−13.1 ± 3.7%, *p* = 0.007), CBP (−39.2 ± 15.2%, *p* = 0.03), and ARC (−33.1 ± 8.1%, *p* = 0.003) were significantly decreased.

Consistent with impaired downstream signaling, phosphorylation of key pathway components was also reduced, including p‐ERK1/2 (−33.8 ± 9.5%, *p* = 0.007), p‐Elk‐1 (−42.6 ± 11.3%, *p* = 0.005), and p‐CREB (−35.1 ± 14.2%, *p* = 0.03), relative to their respective total protein levels. Correspondingly, the p‐CREB/CREB, p‐Elk‐1/Elk‐1, and p‐ERK/ERK ratios were significantly reduced (Figure 2D), indicating diminished signaling activation despite the presence of residual protein. Together, these findings demonstrate a broad suppression of BDNF–TrkB–ERK–CREB signaling, consistent with impaired synaptic plasticity and reduced transcription of activity‐regulated genes such as ARC.

### 3.3 Immunofluorescence suggests reductions in BDNF, CBP, and ARC expression occur predominantly in neurons of the hippocampal CA1 region

**FIGURE 3:**
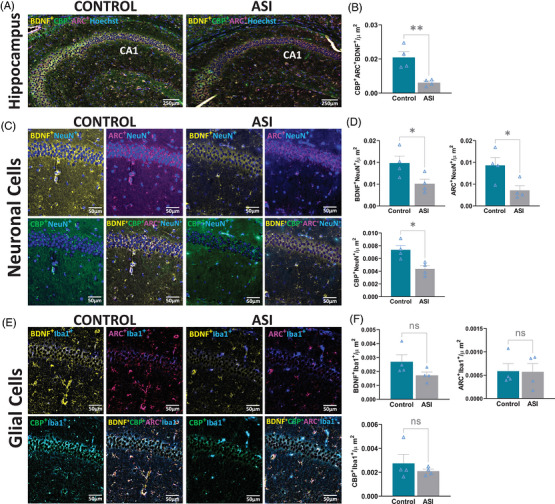
Immunofluorescence analysis of hippocampal BDNF, CBP, and ARC expression reveals predominantly neuronal reductions following ASI. (A) Representative multiplex FlexAble immunofluorescence images showing regional BDNF, CBP, and ARC expression in the hippocampal CA1 region of control and ASI mice. Representative confocal images show BDNF (yellow), CBP (green), ARC (magenta), NeuN and Iba1 (cyan), and Hoechst nuclear staining (blue). (B) Corresponding quantification of BDNF^+^ CBP^+^ ARC^+^ triple‐positive cells showing significant reduction following ASI. (C–D) Representative images and corresponding quantification of neuronal BDNF^+^ NeuN^+^, ARC^+^ NeuN^+^, and CBP^+^ NeuN^+^ cells demonstrating significant reductions in ASI mice. (E–F) Representative images and corresponding quantification of BDNF^+^ Iba1^+^, ARC^+^ Iba1^+^, and CBP^+^ Iba1^+^ microglial cells showing no significant differences between groups. Scale bars: 250 µm (A) and 50 µm (C–F). Immunofluorescence analyses were performed in *n* = 4 mice per group. Cell densities were quantified in CA1 and expressed as cells/µm^2^. Data are presented as mean ± SEM. Statistical comparisons were performed using two‐tailed unpaired *t*‐tests. *p < 0.05, **p < 0.01, ns = not significant.

Multiplex FlexAble immunofluorescence imaging demonstrated robust co‐localization of BDNF, CBP, and ARC within the hippocampal CA1 region of control animals, consistent with intact regional expression of activity‐regulated plasticity proteins (Figure 3A,B). In contrast, ASI exposure resulted in disrupted spatial distribution of these proteins and a significant reduction in BDNF^+^ CBP^+^ ARC^+^ triple‐positive cells within the CA1 region (−0.01 ± 0.002 cells/µm^2^, *p* = 0.005), indicating loss of coordinated expression of key neurotrophic and transcriptional regulators.

Cell‐type‐specific multiplex analyses demonstrated that these alterations predominantly impacted neurons. Quantification of double‐positive neuronal cells revealed significant reductions in BDNF^+^ NeuN^+^ cells (−0.0047 ± 0.001 cells/µm^2^, *p* = 0.04), ARC^+^ NeuN^+^ cells (−0.0057 ± 0.0020 cells/µm^2^, *p* = 0.03), and CBP^+^ NeuN^+^ cells (−0.0029 ± 0.00085 cells/µm^2^, *p* = 0.0129) in ASI animals compared with controls (Figure 3C,D). These findings suggest reduced neuronal expression of proteins involved in activity‐dependent transcription and synaptic regulation.

In contrast, analysis of microglial populations did not find significant differences in BDNF^+^ Iba1^+^ cells (−0.00097 ± 0.00055 cells/µm^2^, *p* = 0.12), ARC^+^ Iba1^+^ cells (*p* = 0.95), or CBP^+^ Iba1^+^ cells (−0.00065 ± 0.00075 cells/µm^2^, *p* = 0.42) between ASI and control groups (Figure 3E,F), although modest downward trends were observed for BDNF and CBP. These results suggest that ASI‐associated alterations in BDNF, CBP, and ARC expression may occur primarily within neuronal populations rather than microglial cells.

Collectively, these findings suggest that ASI induces region‐ and cell‐type‐specific reductions in BDNF, CBP, and ARC expression that are predominantly localized to hippocampal neurons, supporting a neuronal basis for ASI‐associated disruption of molecular pathways involved in hippocampal function.

### 3.4 ASI induces structural synaptic degeneration and delirium‐like behavioral deficits

**FIGURE 4:**
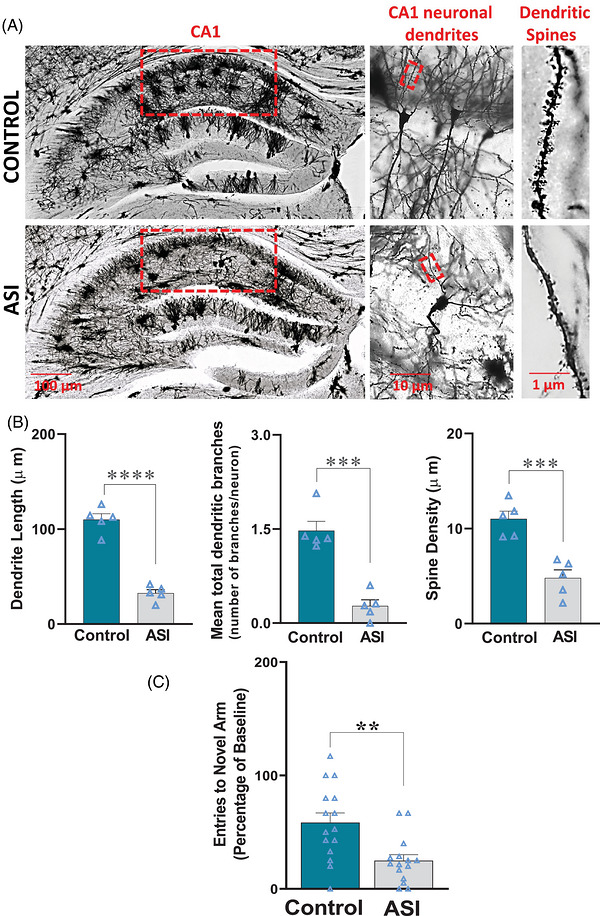
Golgi‐Cox staining analysis of hippocampal neurons and associated memory deficits. (A) Representative Golgi‐stained CA1 pyramidal neurons from control and ASI mice. Higher‐resolution images are shown with scale bars ranging from 100 to 1 µm. (B) Quantitative analysis of dendritic morphology, including length of the longest primary dendrite (µm), average number of dendritic branches, and dendritic spine density (spines/µm). *n* = 5 mice per group, with the five most structurally intact neurons analyzed per mouse. (C) Y‐maze test assessing spatial recognition memory. ASI mice showed reduced entries into the novel arm compared with controls. *n* = 15 mice per group. Statistical comparisons were performed using two‐tailed unpaired *t*‐tests. Data are presented as mean ± SEM. *p < 0.05, **p < 0.01 and ***p < 0.0001.

Golgi‐Cox morphological analysis revealed significant reductions in dendritic architecture and spine density in hippocampal CA1 neurons in ASI mice (Figure 4A,B). Quantitative analysis demonstrated marked reductions in total dendritic length (−77.38 ± 7.132 µm, *p* < 0.0001), branch complexity (−1.199 ± 0.171, *p* = 0.0002), and spine density (−6.213 ± 1.184 spines/µm, *p* = 0.0008) in CA1 neurons of ASI mice. The loss of fine dendritic spines’ structural correlates of synaptic strength and learning capacity indicates microstructural synaptic injury consistent with delirium‐associated cognitive fragility.

The cognitive behavioral deficits mirrored delirium‐like attentional disruption (Figure 4C). In the Y‐maze task, ASI mice made significantly fewer entries into the novel arm (33.5 ± 9.9, *p* = 0.0023), reflecting impaired exploratory drive, working memory, and attentional orientation behavioral domains selectively impaired in human delirium. The behavioral phenotype is consistent with structural and molecular findings, strengthening the translational relevance of the ASI model.

### 3.5 Epigenetic repression signature: a pronounced loss of histone acetylation accompanied by changes in DNA methylation was observed following ASI

**FIGURE 5:**
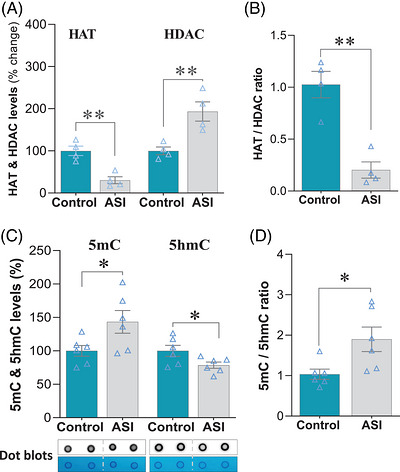
Effects of anesthesia, surgery, and ICU‐like stress on global histone acetylation and DNA methylation.(A) HAT and HDAC activity in Control and ASI mice. (B) HAT/HDAC activity ratio. (C) Global levels of 5‐methylcytosine (5mC) and 5‐hydroxymethylcytosine (5hmC) assessed by dot blot analysis, with representative dot blots shown below. (D) Ratio of 5mC to 5hmC. Data are presented as mean ± SEM, with each dot representing an individual animal (*n *= 4 mice per group for panels A–B; *n* = 6 mice per group for panels C–D). Statistical comparisons were performed using two‐tailed unpaired *t*‐tests. *p < 0.05, **p < 0.01.

Enzymatic activity profiling (*n* = 4 per group; Figure 5A,B) demonstrated a 52% reduction in HAT activity (*p* = 0.007) and a 93% increase in HDAC activity (*p* = 0.009), resulting in a significantly reduced HAT/HDAC ratio (−0.82 ± 0.14, *p* = 0.001).

Consistent with these changes, ELISA‐based quantification of global DNA methylation (*n* = 3 per group) revealed a non‐significant increase in 5mC levels (0.017 ± 0.01, *p* = 0.23, ns) and a significant reduction in 5hmC (0.0098 ± 0.003, *p* = 0.03; Figure S1A), resulting in an increased 5mC/5hmC ratio (1.72 ± 0.37, *p* = 0.01; Figure S1B).

In parallel, global DNA methylation was evaluated by dot blot analysis in an expanded cohort (*n* = 6 per group; Figure 5C,D). Dot blot analysis confirmed a significant increase in global 5mC levels (43.34 ± 18, *p* = 0.043), together with a significant decrease in 5hmC (21.51 ± 9, *p* = 0.046), yielding an increased 5mC/5hmC ratio (0.86 ± 0.32, *p* = 0.02). These results were consistent with ELISA findings and provide independent validation of the observed DNA methylation changes. Together, these results demonstrate coordinated alterations in histone acetylation and DNA methylation markers following ASI.

### 3.6 HDAC inhibition by SAHA attenuates ASI‐induced hippocampal epigenetic, molecular, behavioral, and structural alterations

**FIGURE 6:**
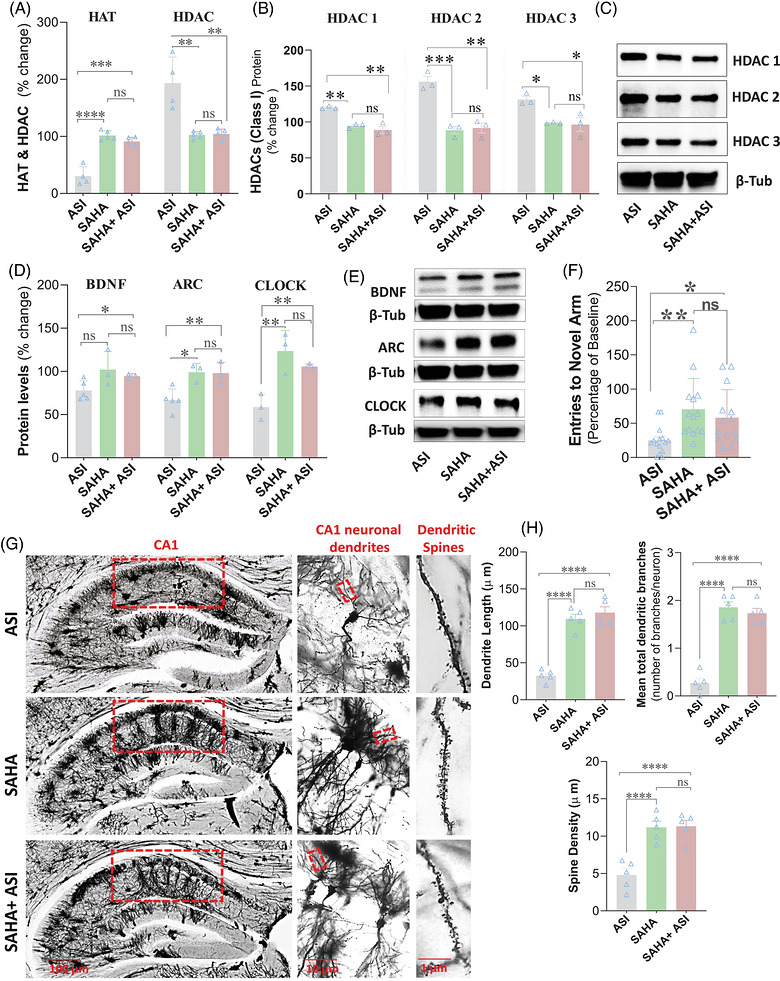
HDAC inhibition with SAHA mitigates ASI‐associated alterations in hippocampal plasticity, circadian signaling, and memory performance. (A) HAT and HDAC activity in ASI and SAHA+ASI groups. (B–C) Class I HDAC isoforms (HDAC1‐3), showing protein expression levels (B) and representative Western blots (C). (D–E) Protein expression levels of BDNF, ARC, and CLOCK, with quantification (D) and representative immunoblots (E). (F) Y‐maze assessment of spatial novelty recognition. (G) Representative Golgi‐stained hippocampal CA1 neurons from ASI, SAHA, and SAHA+ASI mice (scale bars = 100 µm, 10 µm, and 1 µm). (H) Quantification of dendritic length, total branch number, and spine density. Sample sizes were as follows: HAT/HDAC activity assays (*n* = 4‐5 mice per group); protein expression analyses (*n* = 3‐5 mice per group); Y‐maze testing (*n* = 15 mice per group); Golgi analyses (*n* = 5 mice per group). Data are presented as mean ± SEM. Statistical comparisons were performed using two‐tailed unpaired *t*‐tests or one‐way ANOVA with Tukey's multiple comparisons test, as appropriate. *p < 0.05 and ** *p* < 0.01.

SAHA treatment significantly attenuated ASI‐induced alterations in hippocampal histone acetylation. Compared with ASI alone, the SAHA + ASI group showed a significant increase in HAT activity (60.83%, *p* = 0.0001) and a marked reduction in HDAC activity (89.33%, *p* = 0.003) (Figure 6A).

Consistent with these changes in HAT and HDAC activity, protein expression levels of HDAC1 (31.1%, *p* = 0.001), HDAC2 (35.9%, *p* = 0.001), and HDAC3 (34.8%, *p* = 0.01) were significantly decreased in the SAHA + ASI group compared with ASI alone (Figure 6B,C). Protein expression analysis further demonstrated increased levels of several neuroplasticity and circadian‐related proteins following SAHA treatment. In the SAHA + ASI group, expression of BDNF (16.6%, *p* = 0.05), ARC (31.2%, *p* = 0.01), and CLOCK (31.2%, *p* = 0.01) was significantly higher compared with ASI alone (Figure 6D,E).

Behavioral analysis showed that, compared with ASI mice, the SAHA + ASI group exhibited a greater number of entries into the novel arm in the Y‐maze task (33.2%, *p* = 0.04) (Figure 6F). Structural analysis using Golgi‐Cox staining (Figure 6G) demonstrated improved dendritic architecture in SAHA‐treated animals. Relative to ASI alone, the SAHA + ASI group showed increased total dendritic length (85.22 µm, *p* = 0.0001), mean dendritic branch number (1.45 branches per neuron, *p* = 0.0001), and spine density (6.5 spines/µm, *p* = 0.0003) (Figure 6H).

### 3.7 Network enrichment identifies BDNF and core CLOCK genes as hub regulators of POD‐associated pathways

**FIGURE 7:**
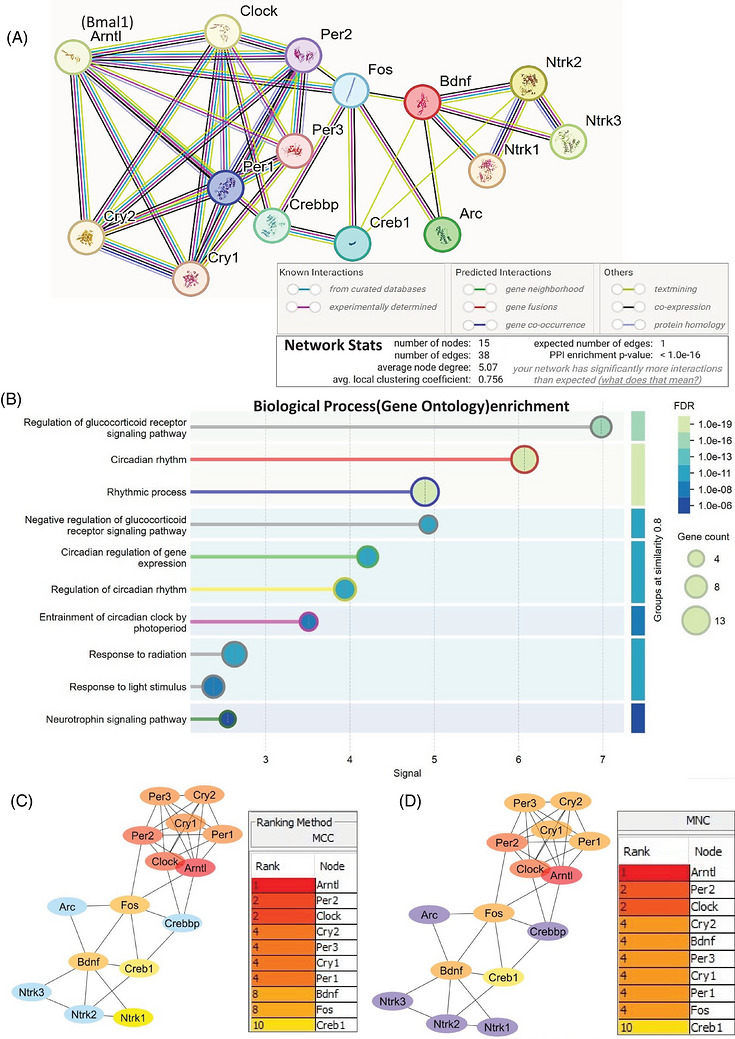
Network and enrichment analysis of genes involved in circadian rhythm and BDNF/TrkB signaling. (A) PPI network generated using STRING, illustrating interactions among 15 genes. Network enrichment was significant (*p* < 1.0 × 10^−^^1^ ^6^). (B) Gene Ontology (GO) biological process enrichment analysis of network‐associated genes. Bubble size represents gene count; color indicates FDR‐adjusted significance. (C–D) Hub gene identification using CytoHubba in Cytoscape based on MCC and MNC algorithms. Top‐ranked hub genes include Arntl (BMAL1), Per2, CLOCK, Cry2, and BDNF, highlighting close integration of circadian and neuroplastic signaling pathways.

STRING‐based PPI reconstruction revealed a densely interconnected module linking neurotrophin signaling components with circadian regulators (Figure 7A). The network included BDNF, Ntrk1, Ntrk2, Ntrk3, Fos, and Creb1, together with circadian genes CLOCK, Arntl (BMAL1), Per1, Per2, Per3, Cry1, and Cry2. The significantly enriched interaction density (PPI enrichment *p* < 1.0 × 10^−^^1^ ^6^) indicates that these genes form a highly interconnected functional network rather than occurring together by chance.

Gene Ontology enrichment analysis (Figure 7B) highlighted pathways related to circadian regulation of transcription, neurotrophin signaling, and glucocorticoid receptor signaling, processes that are relevant to physiological stress responses and circadian regulation. Enrichment of terms such as “entrainment of circadian CLOCK by photoperiod” and “negative regulation of glucocorticoid signaling” suggests potential interactions between circadian regulation and stress‐responsive transcriptional pathways.

Hub gene identification using maximal clique centrality (MCC) and maximum neighborhood component (MNC) algorithms (Figure 7C,D) consistently ranked Arntl, Per2, CLOCK, Cry2, and BDNF among the highest‐centrality nodes in the network. The co‐identification of CLOCK/BMAL1 circadian regulators and BDNF, a key neurotrophin signaling molecule, indicates that circadian transcriptional regulators and neurotrophic signaling components occupy central positions within the reconstructed interaction network.

### 3.8 Integrative model: ASI induces delirium‐like cognitive impairments via epigenetic silencing of the BDNF–ARC–circadian loop

**FIGURE 8:**
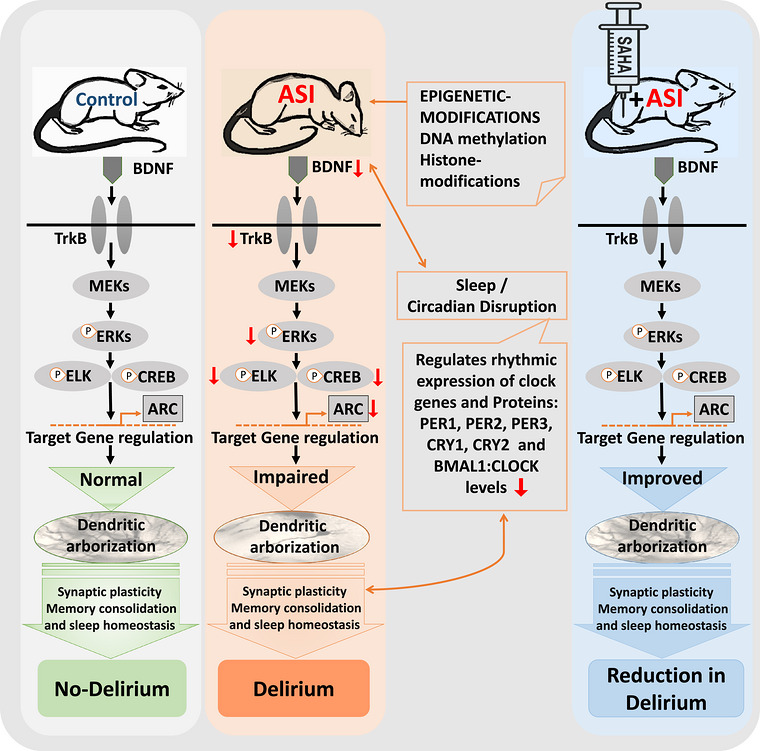
Schematic illustrating how perioperative stress disrupts neuroplastic and circadian signaling through epigenetic dysregulation, and how HDAC inhibition mitigates these alterations. Under physiological conditions, BDNF–TrkB signaling engages the MEK/ERK–CREB pathway to support activity‐dependent gene expression and synaptic integrity, while circadian transcription factors (BMAL1:CLOCK) rhythmically gate neuroplastic programs. Exposure to anesthesia, surgery, and ICU‐like stress (ASI) is associated with epigenetic alterations, reduced neuroplastic and circadian gene expression, and disrupted dendritic architecture, coinciding with delirium‐like phenotypes. HDAC inhibition with SAHA restores histone acetylation, reengages neuroplastic and circadian signaling, and is associated with reduced delirium‐like deficits.

Our proposed integrative mechanistic model (Figure 8) illustrates how ASI induces coordinated epigenetic remodeling that suppresses BDNF–TrkB–CREB–ARC signaling, resulting in dendritic degeneration and memory impairment. In parallel, ASI disrupts circadian gene expression, so that sleep–wake fragmentation and molecular CLOCK dysfunction act synergistically with synaptic injury to promote delirium‐associated cognitive decline. Together, these data suggest that neuroplasticity and circadian entrainment represent mechanistically coupled pathways rather than independent regulatory systems.

When considered in the context of the epigenetic repression signature observed in ASI‐exposed hippocampi (Figure 5), our network topology data suggest that loss of chromatin accessibility at CLOCK–BDNF hub loci may disrupt transcriptional regulation of neurotrophin signaling. Circadian transcription factors (CLOCK/BMAL1) regulate rhythmic transcriptional programs that include neurotrophin signaling components. Under ASI conditions, this regulatory balance appears disrupted, leading to blunted neuroplasticity signaling and heightened vulnerability to POD.

Consistent with this model, pathway interrogation revealed a significant attenuation of the BDNF–TrkB–MEK/ERK–CREB axis under ASI conditions (Figure 8). Relative to control animals, ASI‐exposed cohorts exhibited reduced BDNF, reduced TrkB expression, and decreased ERK phosphorylation, accompanied by diminished ELK and CREB activation. This downstream repression was associated with marked suppression of ARC expression, consistent with impaired dendritic arborization and loss of activity‐dependent synaptic plasticity programs.

Circadian regulatory components were simultaneously transcriptionally attenuated following ASI exposure. In line with our previous ASI observations,^9^ expression of Per1, Per2, Per3, Cry1, Cry2, and the BMAL1:CLOCK complex was significantly reduced, indicating a breakdown in rhythmic transcriptional control. The concurrent suppression of BDNF pathway activation and circadian gene expression identifies a decoupling of plasticity‐associated transcription from circadian regulation.

Integration of these molecular findings with the epigenetic data (Figure 5) indicates that ASI‐induced histone hypoacetylation and hypermethylated chromatin states coincide with transcriptional silencing of both BDNF–TrkB signaling and circadian CLOCK regulators, defining a coordinated failure of neuroplastic and oscillation‐keeping transcriptional networks. When administered 1 h prior to ASI and again 12 h after completion of the simulated ICU phase, SAHA partially mitigated ASI‐induced hippocampal epigenetic dysregulation and improved downstream molecular, behavioral, and structural phenotypes. SAHA treatment improved histone acetylation balance, re‐engaged BDNF–TrkB–CREB–ARC signaling, and improved dendritic architecture and cognitive performance (Figure 6).

## 4 DISCUSSION

This study demonstrates that ASI in aged mice produce a coordinated disruption of hippocampal BDNF–TrkB–CREB–ARC signaling, accompanied by epigenetic repression and dendritic degeneration. Together with our prior findings of electroencephalogram fragmentation, circadian dysregulation, and delirium‐like cognitive deficits following anesthesia and ICU‐like stress,^8^, ^9^ these data establish a unified mechanistic framework linking perioperative stress to neuroplastic failure, circadian misalignment, and heightened cognitive vulnerability. The observed downregulation of BDNF exons I–IXA, TrkB, CREB, and ARC reflects a collapse of activity‐dependent neurotrophic signaling an axis essential for dendritic maintenance, spine stability, and memory encoding.

Reductions in CREB and ERK phosphorylation, diminished ARC expression, and loss of dendritic complexity in CA1 pyramidal neurons provide convergent molecular and structural evidence that ASI induces a transient synaptic disconnection state resembling the acute cognitive fragmentation observed in POD and AD.

Consistent with these molecular and structural findings, multiplex immunofluorescence demonstrated that ASI‐induced reductions in BDNF, CBP, and ARC were preferentially localized to hippocampal neurons, with limited apparent co‐localization within Iba1‐positive microglial populations. This distribution is consistent with the well‐established neuronal enrichment and activity‐dependent roles of BDNF and ARC, as well as the critical role of CBP in neuronal transcriptional regulation of memory‐related plasticity pathways.^50^, ^51^, ^52^ These observations are also consistent with prior Perioperative Neurocognitive Disorders (PND)/POD studies demonstrating impaired hippocampal synaptic plasticity following anesthesia and surgery.^53^, ^54^ Together, these findings suggest that ASI‐associated reductions in BDNF, CBP, and ARC‐related signaling arise primarily within neuronal compartments, supporting a predominantly neuronal basis for the disruption of hippocampal plasticity pathways in this model.

This study does not include longitudinal cognitive follow‐up or direct assessment of AD‐related neuropathology and therefore is not meant to establish progression to dementia. Rather, we propose that the changes we observed here define mechanistic substrates of vulnerability that warrant further investigation. Clinically, POD is associated with an increased risk of long‐term cognitive decline and dementia.^55^, ^56^, ^57^ Within that established epidemiological context, our findings identify molecular, epigenetic, and structural alterations in the aged hippocampus that overlap with pathways implicated in cognitive vulnerability and neurodegeneration. These alterations provide a potential biological framework through which delirium‐related vulnerability could contribute to longer‐term decline.

Delirium is a distributed network disorder involving prefrontal, hippocampal, and subcortical circuits.^33^, ^34^ The hippocampus was selected as an initial region of interest because of its role in attention and memory^35^, ^36^ and its well‐characterized BDNF–TrkB–CREB–ARC cascade and epigenetic regulation of plasticity.^58^, ^59^ Emerging experimental evidence supports hippocampal network dysfunction in POD, demonstrating that anesthesia and surgical stress alter hippocampal neural activity contributing to delirium‐like behavior in aged mice.^60^ These findings are in line with our observation that ASI disrupts hippocampal plasticity signaling and epigenetic regulation, supporting the hippocampus as a critical hub within a broader delirium‐related network. Thus, the hippocampal alterations reported here are interpreted as node‐level manifestations within a distributed network rather than isolated regional pathology.

ASI elicited coordinated epigenetic alterations characterized by reduced HAT activity, increased HDAC activity, and a reduced HAT/HDAC ratio. Increased global 5mC together with reduced 5hmC resulted in an elevated 5mC/5hmC ratio, consistent with a transcriptionally restrictive chromatin state. This profile parallels stress and neurodegeneration models in which histone hypoacetylation and DNA hypermethylation suppress neuronal plasticity genes.^25^, ^40^, ^61^ Selective suppression of CREB‐responsive BDNF promoters is consistent with reduced activity‐dependent transcription rather than global transcriptional failure.^62^, ^63^

Circadian dysregulation also appears to be intertwined with epigenetic repression. Network analysis identified CLOCK, ARNTL/BMAL1, Per2, and Cry2 as central hubs alongside BDNF, underscoring the coupling between circadian transcription and neuroplastic signaling. Under physiological conditions, BDNF expression and circadian oscillations are synchronized to support synaptic homeostasis.^64^ ASI disrupted this coordination, likely through reduced chromatin accessibility. Because the CLOCK–BMAL1 complex possesses intrinsic HAT activity, reduced acetylation may dampen circadian amplitude while suppressing neurotrophic transcription,^26^, ^65^ providing a mechanistic explanation for sleep–wake fragmentation observed in perioperative delirium and early AD.^23^, ^66^ These findings identify chromatin remodeling as a shared regulatory axis linking neuroplastic and circadian vulnerability in aging brains exposed to perioperative stress.

HDAC inhibition with SAHA attenuated ASI‐induced epigenetic, behavioral, and structural deficits, indicating that chromatin repression is mechanistically linked to impaired plasticity and circadian regulation. SAHA was administered prophylactically and used as a pharmacological probe rather than a definitive therapeutic strategy; restoration of chromatin accessibility was accompanied by recovery of BDNF, ARC, and CLOCK expression and normalization of hippocampus‐dependent cognition, supporting mechanistic proof of principle. These findings support the idea that delirium‐like deficits arise from reversible synaptic dysfunction. Future studies using more selective HDAC inhibitors, including HDAC1/3‐preferential compounds (e.g., Entinostat/MS‐275) or HDAC2‐biased inhibitors, may help define the specific HDAC isoforms contributing to ASI‐induced chromatin repression while potentially reducing off‐target effects.

BDNF and ARC are central regulators of dendritic spine stability and memory encoding, while CLOCK integrates synaptic activity with circadian timing. Their coordinated recovery following HDAC inhibition further supports the concept that epigenetic tone regulates shared transcriptional programs governing neuroplasticity and circadian homeostasis.

Delirium is among the strongest clinical predictors of future dementia, and accumulating evidence suggests that delirium episodes may accelerate amyloid beta and tau pathology while amplifying neuroinflammation.^67^, ^68^ By collapsing neuroplastic and circadian signaling, ASI may prime hippocampal networks for maladaptive responses to subsequent pathological stressors. Thus, perioperative chromatin dysregulation may represent an early vulnerability mechanism intersecting with pathways implicated in AD and related dementias, without establishing causal progression. Emerging evidence indicates that molecular and epigenetic alterations associated with delirium and perioperative neurocognitive disorders engage biological pathways central to neurodegenerative susceptibility, including synaptic dysfunction and epigenetic regulation via DNA methylation and histone modification.^57^, ^67^, ^69^

Notably, SAHA was administered prophylactically; whether delayed chromatin modulation can reverse established delirium phenotypes remains unknown. More selective HDAC inhibitors, particularly HDAC1/3‐preferential compounds such as entinostat (MS‐275) or HDAC2‐biased inhibitors, may therefore represent promising next‐generation approaches for targeted epigenetic modulation. Simultaneously, non‐pharmacological interventions known to influence chromatin accessibility, including environmental enrichment, circadian/light optimization, and structured rehabilitative stimulation, may represent complementary strategies for future investigation.^70^, ^71^, ^72^, ^73^, ^74^, ^75^

Our data support integrative prevention strategies targeting molecular and behavioral domains. Pharmacological modulation of chromatin remodeling combined with circadian stabilization strategies may enhance synaptic resilience. Peripheral BDNF levels, 5hmC/5mC ratios, and CLOCK‐gene expression profiles may serve as candidate biomarkers for delirium risk and recovery monitoring. Our perioperative stress model^8^, ^9^ provides a platform for preclinical testing linking epigenetic state, synaptic structure, circadian disruption, and behavior.

Certain limitations should be acknowledged. The findings were derived from aged male mice and therefore may not fully generalize to females, given known sex‐specific differences in neuroinflammatory responses, synaptic plasticity, and vulnerability to delirium and cognitive decline. Epigenetic analyses were performed on bulk hippocampal tissue and therefore reflect global rather than cell‐type‐specific effects. Moreover, behavioral, molecular, and epigenetic endpoints were obtained from two independent animal cohorts, precluding individual‐level correlations. ELISA‐based DNA methylation analyses were limited by sample size; however, key findings were reproduced in a larger cohort using quantitative dot blot analyses, supporting the robustness of the observed methylation changes. Behavioral assessment was limited to the Y‐maze task and therefore may not fully capture the multidimensional and fluctuating nature of delirium‐like behavior. In addition, classification of delirium‐like behavior based on Z‐scores derived from a single behavioral assay may not fully capture the complex nature of the syndrome. Gene expression analyses were performed on pooled hippocampal samples and are therefore interpreted as group‐level pathway signatures associated with perioperative stress exposure rather than direct individual‐level molecular and behavioral associations, which may obscure inter‐individual variability.

We acknowledge that no single assay fully captures the clinical spectrum of delirium. ICU‐like environmental stress is intentionally incorporated into the ASI paradigm to enhance translational relevance, as perioperative and ICU stressors cannot be fully separated from delirium in clinical settings. Finally, while our immunohistochemistry data suggest a prominent role of neurons in the disruption of the BDNF–ARC signaling pathway, additional cell‐type‐specific epigenomic approaches are needed to dissect the contributions of neurons versus other cell populations. Future studies incorporating both sexes, longitudinal designs, multiregion analyses, and cell‐type‐specific approaches will be required to better characterize how early perioperative epigenetic dysregulation shapes network vulnerability and long‐term cognitive trajectories.

## 5 CONCLUSION

Exposure to ASI in aged mice induces epigenetically mediated disruption of hippocampal neuroplasticity, characterized by repression of the BDNF–TrkB–CREB–ARC axis, dendritic degeneration, circadian dysregulation, and cognitive impairment. Multiplex immunofluorescence analyses of the hippocampal CA1 region further indicate that ASI‐induced reductions in BDNF, CBP, and ARC expression occur predominantly in neuronal populations, supporting a neuronal basis for ASI‐associated disruption of hippocampal neuroplasticity. Central to this process is a shift toward a transcriptionally restrictive chromatin state marked by reduced histone acetylation, increased HDAC activity, and altered global DNA methylation, which may suppress activity‐dependent neurotrophic signaling and destabilize synaptic architecture.

HDAC inhibition with SAHA attenuated these molecular, structural, and behavioral abnormalities, demonstrating that perioperative synaptic and circadian dysfunction is mechanistically linked to chromatin repression and may be biologically tractable. These findings position chromatin remodeling as a central regulatory mechanism underlying delirium‐like cognitive vulnerability. They also identify epigenetically dysregulated neuroplastic and circadian signaling networks as convergent mechanistic pathways engaged by perioperative stress. Notably, these alterations intersect with pathways implicated in AD and related dementias; however, future studies will be needed to determine whether these shared mechanisms contribute to the progression from delirium to dementia.

## CONFLICT OF INTEREST STATEMENT

The authors declare no conflicts of interest. Author disclosures are available in the Supporting Information.

## Consent Statement

Not applicable. This study did not involve human participants or human‐derived samples.

## Supporting information



Supporting Information:

Supporting Information

Supporting Information:

Supporting Information

Supporting Information
