# Supplementary material for: Epigenetically driven impairment of BDNF–ARC signaling contributes to circadian and cognitive disarray in a mouse model of postoperative delirium
Source: Alzheimers Dement. 2026 Jun 15;22(6):e71556. doi: 10.1002/alz.71556 (PMC13269003; doi:10.1002/alz.71556)
Supplement: Supplementary file 1 — Supporting Information: [file ALZ-22-e71556-s005.docx]

**Supplementary Table S1. Experimental group allocation and animal numbers. All experiments were conducted using aged male mice. Experimental group allocation and animal numbers for each assay are summarized for Cohort Group 1 (ASI model validation cohort) and Cohort Group 2 (independent experimental cohort used for mechanistic analyses).**

***** Behavioral data from Cohort Group 1 were previously published by our laboratory (Illendula et al., 2020; Dulko et al., 2023) [8,9] and are not re-presented in the current manuscript.

**^#^** Global DNA methylation (5mC/5hmC) levels were assessed using two complementary methods: ELISA (n = 3 mice per group) and dot blot analysis (n = 6 mice per group).

**‡** HAT/HDAC activity assays were performed with n = 4–5 mice per group, and HDAC1–3 protein expression analyses were performed with n = 3 mice per group in Cohort Group 2.

| **Cohort Group 1**  Experiment | Control (*n*) | ASI (*n*) | | |  |
| --- | --- | --- | --- | --- | --- |
| Behavior* | 24 | 24 | | |  |
| qRT-PCR (pooled) | 3 | 3 | | |  |
| 5mC/5hmC**^#^** | 3-6 | 3-6 | | |  |
| Immunofluorescence | 4 | 4 | | |  |
| **Cohort Group 2**  Experiment | Control (*n*) | ASI (*n*) | SAHA (*n*) | SAHA+ASI (*n*) | |
| Behavior  Golgi staining | 15  5 | 15  5 | 15  5 | 15  5 | |
| Target protein expression | 5 | 5 | 5 | 5 | |
| HAT/HDAC activity and HDAC1–3 protein expression**^‡^** | 3-4 | 3-4 | 3-5 | 3-5 | |
