# Supplementary material for: Epigenetically driven impairment of BDNF–ARC signaling contributes to circadian and cognitive disarray in a mouse model of postoperative delirium
Source: Alzheimers Dement. 2026 Jun 15;22(6):e71556. doi: 10.1002/alz.71556 (PMC13269003; doi:10.1002/alz.71556)
Supplement: Supplementary file 2 — Supporting Information [file ALZ-22-e71556-s004.docx]

**Supplementary Table S2**: List of the target gene primer sequences used in qPCR expression analysis. Primers were purchased from Sigma Aldrich or from Bio-Rad Laboratories, Inc. USA.

| Primer | Forward Primer (5' → 3') | Reverse Primer (5' → 3') |
| --- | --- | --- |
| BDNF exon I | TTACCTTCCTGCATCTGTTGG | GTCATCACTCTTCTCACCTGG |
| BDNF exon IIa | GAGAGCAGAGTCCATTCAGC | CCTTCATGCAACCGAAGTATG |
| BDNF exon IIb | AGAGTCAGATTTTGGAGCGG | GTCATCACTCTTCTCACCTGG |
| BDNF exon IIc | GGCTGGAATAGACTCTTGGC | GTCATCACTCTTCTCACCTGG |
| BDNF exon III | GGGCTCCTGCTTCTCAAG | ATGCAACCGAAGTATGAAATAACC |
| BDNF exon IV | AGCTGCCTTGATGTTTACTTTG | CGTTTACTTCTTTCATGGGCG |
| BDNF exon V | AACCATAACCCCGCACAC | ATGCAACCGAAGTATGAAATAACC |
| BDNF exon VI | GGACCAGAAGCGTGACAAC | ATGCAACCGAAGTATGAAATAACC |
| BDNF exon VII | TGAAAGGGTCTGCGGAAC | GTCATCACTCTTCTCACCTGG |
| BDNF exon VIII | CTGATTGCTGAAAATGGTGTCG | AGTTGCCTTGTCCGTGG |
| BDNF exon IXA | AGTTCTAACCTGTTCTGTGTCTG | CGTTTACTTCTTTCATGGGCG |
| *Arc* | CCAGGAGAATGACACCAG | TTCAGGAGAAGAGAGGATG |
| *Ntrk1* (TrkA) | GTAACAGCACATCAAGAGAC | ATCCTCTGGAGCTAATACAG |
| *Ntrk2* (TrkB) | ACGAGACAAACCCAAATTAC | CTTTTGTTCGTAGTATCCCC |
| *Ntrk3* (TrkC) | TTTGACTTTGAGTCTGATGC | CATGATAAAGAGAACCACCAG |
| *Creb1* | TCTAATGAAGAACAGGGAGG | GTCCTTAAGTGCTTTTAGCTC |
| *Gapdh* | Mouse Prime PCR Assay #10025636 (Bio Rad) | |
