# Supplementary material for: Epigenetically driven impairment of BDNF–ARC signaling contributes to circadian and cognitive disarray in a mouse model of postoperative delirium
Source: Alzheimers Dement. 2026 Jun 15;22(6):e71556. doi: 10.1002/alz.71556 (PMC13269003; doi:10.1002/alz.71556)
Supplement: Supplementary file 3 — Supporting Information: [file ALZ-22-e71556-s003.docx]

**Supplementary Table S3.** List of primary antibodies and dilutions used in Western blot analysis.
Secondary antibodies anti-rabbit IgG–HRP (#7074) and anti-mouse IgG–HRP (#7076) were used at a 1:10,000 dilution and were purchased from Cell Signaling Technology, Inc. (USA).

| \| **Protein** \| \| --- \| | \| **Primary antibody dilution** \| \| --- \| | \| **Catalog number and Company** \| \| --- \| |
| --- | --- | --- | --- | --- | --- |
| BDNF | Rabbit monoclonal, 1:2000 | #A4873, ABclonal, Inc. |
| TrkB | Rabbit monoclonal, 1:2000 | #4603, Cell Signaling Technology |
| Pan-Trk | Rabbit monoclonal, 1:2000 | #92991, Proteintech Group |
| ERK1/2 (p44/42 MAPK) | Rabbit polyclonal, 1:3000 | #9102, Cell Signaling Technology |
| Phospho-ERK1/2 (Thr202/Tyr204) | Rabbit monoclonal, 1:3000 | #4370, Cell Signaling Technology |
| ELK1 | Rabbit polyclonal, 1:2000 | #9182, Cell Signaling Technology |
| Phospho-ELK1 (Ser383) | Rabbit polyclonal, 1:2000 | #9181, Cell Signaling Technology |
| CREB | Rabbit monoclonal, 1:2000 | #9197, Cell Signaling Technology |
| Phospho-CREB (Ser133) | Rabbit monoclonal, 1:2000 | #9193, Cell Signaling Technology |
| ARC | Mouse monoclonal, 1:2000 | #SC-17839, Santa Cruz Biotechnology |
| CBP | Rabbit monoclonal, 1:2000 | #7389, Cell Signaling Technology |
| β-tubulin | Mouse monoclonal, 1:10,000 | #66240, Proteintech Group |
| 5mC | Mouse monoclonal (clone 33D3), 1:1000 | #MABE146, Millipore Sigma |
| 5hmC | Mouse monoclonal, 1:1000 | #40900, Active Motif |
| HDAC1/2/3 | Mouse monoclonal, 1:3000 | #9928, Histone Deacetylase (HDAC) Antibody Sampler Kit, Cell Signaling Technology |
