# Supplementary material for: Epigenetically driven impairment of BDNF–ARC signaling contributes to circadian and cognitive disarray in a mouse model of postoperative delirium
Source: Alzheimers Dement. 2026 Jun 15;22(6):e71556. doi: 10.1002/alz.71556 (PMC13269003; doi:10.1002/alz.71556)

Supplementary Figure S1. ELISA-based quantification of global DNA methylation and hydroxymethylation.

(A) Global levels of 5-methylcytosine (5mC) and 5-hydroxymethylcytosine (5hmC) in hippocampal DNA from Control and ASI mice. (B) Ratio of 5mC to 5hmC. Data are presented as mean ± SEM, with each dot representing an individual animal ($n = 3$ mice per group). Statistical comparisons were performed using two-tailed unpaired Student's t-test. * $p < 0.05$, ** $p < 0.01$; ns., not significant.

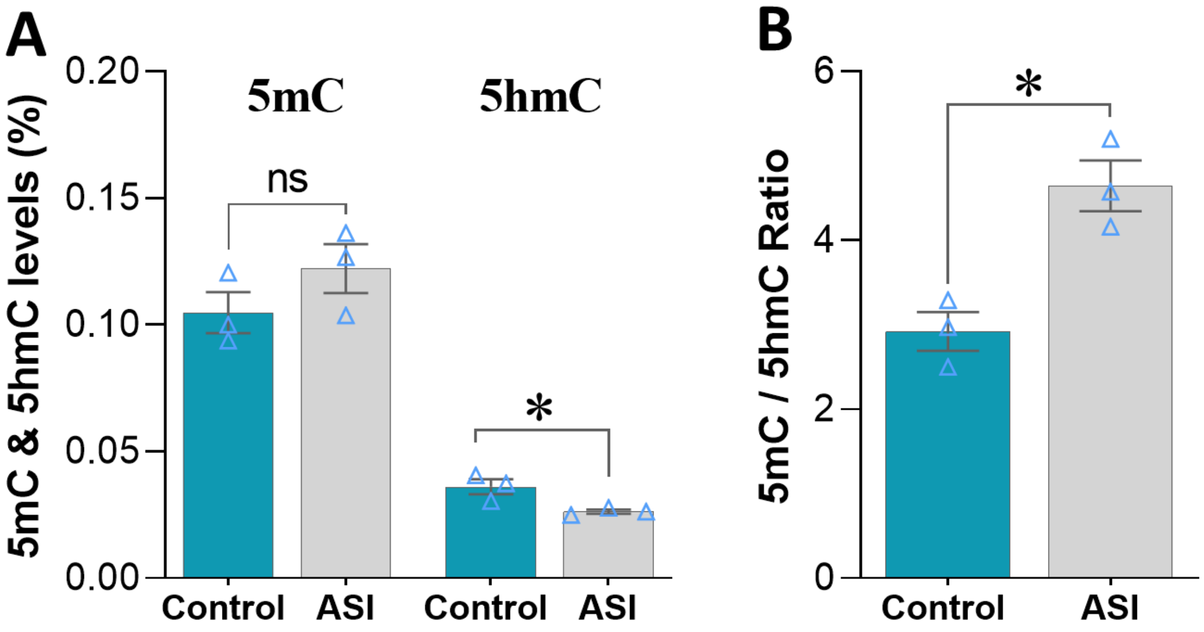

Supplement: Supplementary file 4 — Supporting Information [file ALZ-22-e71556-s002.pdf]
